# Sex Differences in Risk of Adverse Liver Events in Patients With Cirrhosis

**DOI:** 10.1001/jamanetworkopen.2025.23674

**Published:** 2025-07-28

**Authors:** Yu Shi, Xinrong Zhang, Tyler Wong, Taotao Yan, Linda Henry, Ramsey Cheung, Mindie H. Nguyen

**Affiliations:** 1 Division of Gastroenterology and Hepatology, Stanford University Medical Center, Palo Alto, California; 2 State Key Laboratory for Diagnosis and Treatment of Infectious Diseases, National Clinical Research Center for Infectious Diseases, The First Affiliated Hospital, School of Medicine, Zhejiang University, Hangzhou, China; 3 University of California, Santa Barbara; 4 Department of Infectious Diseases, The First Affiliated Hospital of Xi’an Jiaotong University, Shaanxi, China; 5 Division of Gastroenterology and Hepatology, Veterans Affairs Palo Alto Healthcare System, Palo Alto, California; 6 Department of Epidemiology and Population Health, Stanford University Medical Center, Palo Alto, California; 7 Stanford Cancer Institute, Stanford University Medical Center, Palo Alto, California

## Abstract

**Importance:**

Patients with cirrhosis are at high risk of developing adverse liver events. However, data on sex differences are limited.

**Objective:**

To compare risk of adverse liver events between male and female patients with cirrhosis.

**Design, Setting, and Participants:**

This population-based retrospective cohort study included adult patients with cirrhosis who were identified from a US private health insurance claims database (Merative MarketScan Research Databases) from January 1, 2007, to December 31, 2022.

**Exposures:**

Males compared with females.

**Main Outcomes and Measures:**

The main outcome was the incidence of adverse liver events (decompensated cirrhosis [DC], hepatocellular carcinoma [HCC], and liver transplant [LT]). Propensity score matching on age, liver disease etiologies, geographic region, insurance type, specialty type, alcohol use disorder, obesity, baseline status of decompensation, and Charlson Comorbidity Index score was used to balance baseline characteristics of the male and female groups.

**Results:**

The study included 438 706 patients with cirrhosis (mean [SD] age, 56.8 [15.4] years; 50.8% males), with a higher mean (SD) age in males than in females (57.6 [14.3] vs 55.9 [16.4] years). Propensity score matching yielded 169 711 pairs of female and male patients with similar baseline characteristics for subsequent analyses. Males compared with females had a higher incidence (per 1000 person-years) of DC (65.77 [95% CI, 64.74-66.81] vs 55.35 [95% CI, 54.46-56.25]; *P* < .001), HCC (6.98 [95% CI, 6.71-7.27] vs 3.35 [95% CI, 3.17-3.54]; *P* < .001), and LT (10.23 [95% CI, 9.89-10.58] vs 6.27 [95% CI, 6.01-6.52]; *P* < .001). In the Cox proportional hazards regression model, male sex was associated with 16% higher risk of DC (hazard ratio [HR], 1.16 [95% CI, 1.14-1.19]; *P* < .001), 63% of LT (HR, 1.63 [95% CI, 1.54-1.71]; *P* < .001), and 110% of HCC (HR, 2.10 [95% CI, 1.96-2.25]; *P* < .001). Among the major liver disease etiologies, male sex (compared with female sex) was associated with the highest risk of adverse liver events in patients with alcohol-related liver disease including DC (HR, 1.13 [95% CI, 1.08-1.19]; *P* < .001), HCC (HR, 2.40 [95% CI, 2.01-2.88]; *P* < .001), and LT (HR, 1.36 [95% CI, 1.21-1.53]; *P* < .001), followed by metabolic dysfunction-associated steatotic liver disease and hepatitis C virus (HCV) infection, but not in patients with HBV except for those with HCC (HR, 1.60 [95% CI, 1.08-2.36]; *P* = .02).

**Conclusions and Relevance:**

The findings of this cohort study of adult patients with cirrhosis suggest that significant sex differences in liver complication risk exist, which was more pronounced in nonviral (alcohol-related liver disease and metabolic dysfunction-associated steatotic liver disease) compared with viral (HBV and HCV) cirrhosis. Sex disparities should be taken into consideration in future guidelines and programs for disease monitoring, prevention, and treatment of patients with cirrhosis.

## Key Points

**Question:**

Are there differences in the risk of adverse liver events between male and female patients with cirrhosis?

**Findings:**

In this cohort study of 438 706 adult patients with cirrhosis, male sex (compared with female sex) was significantly associated with higher risk of decompensated cirrhosis, hepatocellular carcinoma, and liver transplant..

**Meaning:**

These findings suggest that sex-based approaches should be considered for monitoring and management of adult patients with cirrhosis for potential interventions in the risk of adverse liver events.

## Teaser

This cohort study assesses whether there are differences in the risk of adverse liver events between male and female patients with cirrhosis.

## Introduction

Cirrhosis represents a significant global health burden, with over 1 million individuals dying of its complications annually.^1^ The prevalence of cirrhosis is projected to increase over 50% by 2030,^2^ with metabolic dysfunction-associated steatotic liver disease (MASLD; formerly nonalcoholic fatty liver disease [NAFLD]) and alcohol-associated liver disease (ALD) being the 2 main causes.^2,3,4^

Sex has been described as an important factor influencing the severity of chronic liver disease, with women generally considered to have a more favorable clinical course than men, at least in the earlier stages of chronic liver disease.^5,6,7^ For example, compared with men, women are more likely to achieve spontaneous clearance of hepatitis C virus (HCV) infection^5^ and are less likely to have fibrosis progression caused by viral hepatitis and metabolic dysfunction-associated steatohepatitis (formerly nonalcoholic steatohepatitis [NASH]), particularly women who are premenopausal.^5,6^

However, the disparity of sex in liver disease progression in more advanced stages such as cirrhosis remains inconsistent. Some studies have reported a significantly higher mortality rate of cirrhosis in men compared with women,^8,9^ while others have suggested that, despite lower all-cause mortality, there is no difference in adverse liver mortality risk between males and females.^10^ However, it remains unknown whether this is due to a lack of sex differences in the risk of adverse liver events especially when investigating etiology-specific cirrhosis.

Although sex is an unmodifiable risk factor, understanding sex disparity is essential for optimizing disease management and promoting health equity. To address this gap, we leveraged the Merative MarketScan Research Databases, a commercial health care claims database that includes all 50 US states and 250 million Americans, to examine the association between sex and the risk of adverse liver events, which include decompensated cirrhosis (DC), hepatocellular carcinoma (HCC), and liver transplant (LT) in patients with cirrhosis. We also evaluated these associations across subgroups of patients with cirrhosis by different etiologies.

## Methods

### Patients

In this cohort study, we retrospectively identified adult patients (aged ≥18 years) with cirrhosis from the Merative MarketScan Research Databases, which is housed in the Center for Population Health Sciences at Stanford University,^11,12^ using *International Classification of Diseases, Ninth Revision, Clinical Modification* (*ICD-9-CM*) and *International Statistical Classification of Diseases, Tenth Revision, Clinical Modification *(*ICD-10-CM*) codes for cirrhosis or a clinical decompensating event (ascites, hepatorenal syndrome, hepatic encephalopathy, or variceal bleeding) (eTable 1 in Supplement 1). Only patients with at least 1 inpatient or 2 outpatient diagnoses of cirrhosis or its complications were included. This study adhered to the principals of the Declaration of Helsinki^13^ and was approved by the Institutional Review Boards of Stanford University. The requirement for informed consent was waived because the data were deidentified. The study followed the Strengthening the Reporting of Observational Studies in Epidemiology (STROBE) reporting guideline for cohort studies.

The Merative MarketScan Research Databases comprise approximately 250 million patients from the US from January 1, 2007, to December 31, 2022. The databases link paid claims and encounter (inpatient, outpatient, and outpatient pharmacy) data to detailed patient information across locations and types of health care organizations and over time for approximately 350 private payers to also include certain Medicare Advantage plans. We excluded patients with organ transplants prior to cirrhosis diagnosis or at baseline (within 30 days after diagnosis) and those with HCC and other malignant neoplasms within 5 years before cirrhosis diagnosis, at baseline, or within 6 months after diagnosis.

### Primary Outcomes

The index date was defined as the date of the first cirrhosis diagnosis. The primary study outcomes were defined as the incidence of DC, HCC, LT. DC was defined by the presence of ascites, hepatorenal syndrome, hepatic encephalopathy, and/or variceal bleeding (eTable 1 in Supplement 1). Incident events of DC, HCC, and LT were defined as those occurring 6 months after the study index. DC prior to or within 6 months of the cirrhosis diagnosis date was considered a prevalent event, and these patients were excluded from incidence analyses of DC. Patients were followed up to the occurrence of the primary outcomes (DC, HCC, or LT) or censored at the insurance enrollment end date, the last follow-up date, or the end of the study period (December 31, 2022), whichever came first.

Liver disease etiologies were classified as HCV, HBV, ALD, MASLD, cryptogenic, and other etiologies (eg, autoimmune hepatitis, primary biliary cholangitis, primary sclerosing cholangitis, Wilson diseases, hemochromatosis, and α-1 antitrypsin deficiency) (eTable 1 in Supplement 1). The diagnosis of cryptogenic cirrhosis was made when no specific cause for cirrhosis could be identified. When viral hepatitis coexisted with other liver disease etiologies such as ALD or MASLD, viral hepatitis was considered the primary etiology and categorized as such.

### Statistical Analysis

Continuous variables were represented as mean (SD) or median (IQR) and compared by the *t* test or the Wilcoxon rank sum test as appropriate between the male and female groups. Categorical data were represented as numbers (percentages) and compared by the χ^2^ test. Patients in the male and female groups were matched by propensity score matching (PSM) with a caliper of 0.1 for age, etiologies of cirrhosis, geographic region, insurance type, specialty type (receipt of gastroenterology or infectious disease [GI/ID] specialist care vs not), alcohol use disorder, obesity, baseline status of DC, and Charlson Comorbidity Index score. Variables with a standardized mean difference (SMD) less than 0.1 were considered to be well balanced. Kaplan-Meier estimates were used to estimate the cumulative incidence of DC, HCC, and LT between male and female patients and were compared by the log-rank test. Cox proportional hazards regression models were used to estimate hazard ratios (HRs) and 95% CIs for male sex compared with female sex for the adverse liver event of interest. We also calculated absolute risk differences between females and males. The proportional hazards assumption was tested using the Schoenfeld residual test with no violation of proportionality observed. We performed a sensitivity analysis using inverse probability of treatment weighting to balance the same variables as the main analysis with PSM to assess the robustness of the findings. To detect potential significant unmeasured confounders, we calculated E-values to help assess the degree of residual bias required to nullify the observed associations, with large E-values indicating that considerable confounding would be required to nullify the association and suggesting that the observed association would likely be robust.^14^ We performed subgroup analyses stratified by major liver disease etiology, median age, baseline DC, and the time of cirrhosis diagnosis stratified by the median of the study period (before or in 2014 and after 2014). All analyses were conducted using R, version 4.1.1 (R Project for Statistical Computing). Two-sided tests of significance were used, and *P* < .05 or SMD > 0.1 was considered statistically significant.

## Results

### Baseline Characteristics

**Figure 1.**
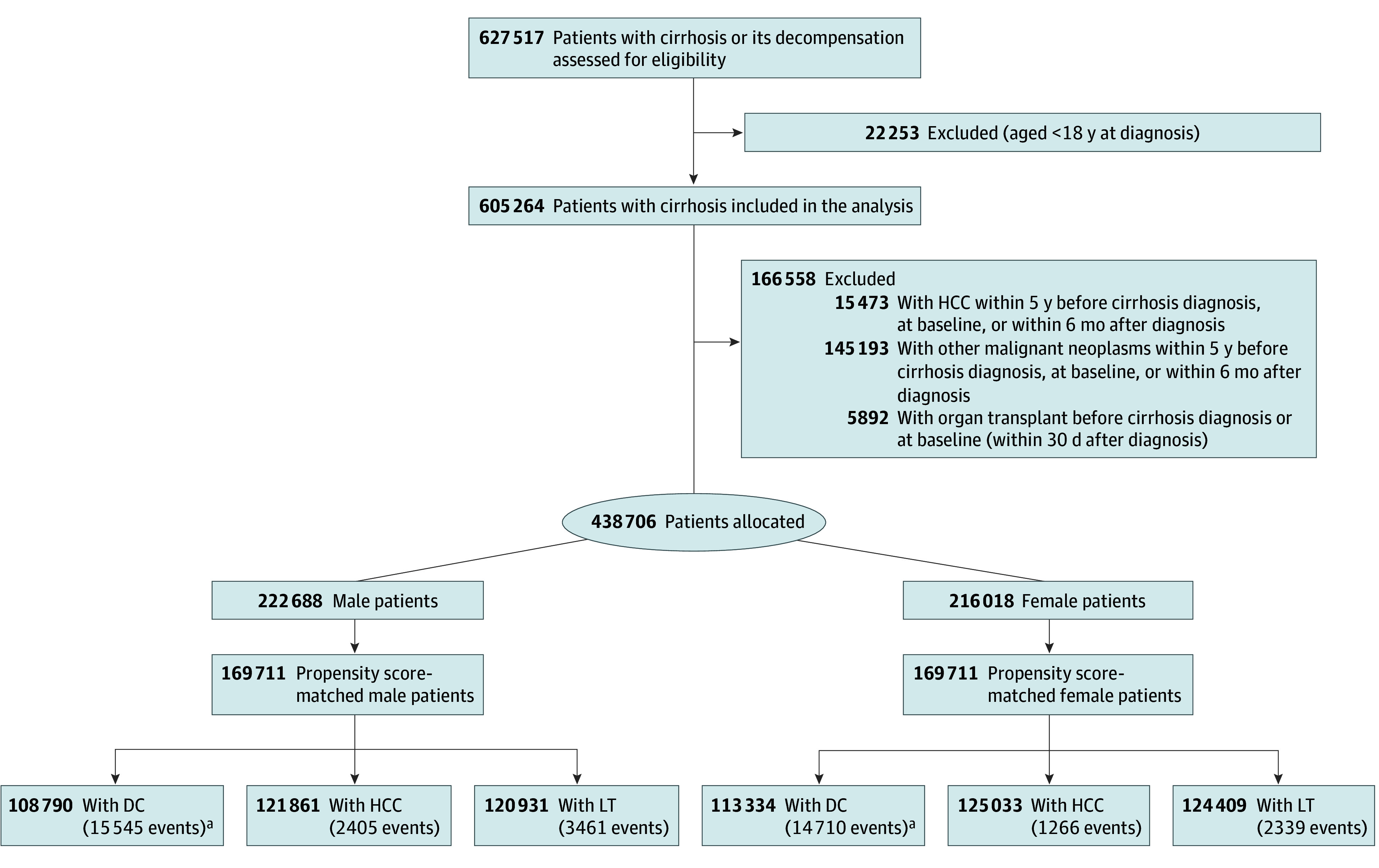
Study Flowchart Adult patients (aged ≥18 years) with cirrhosis were identified from the Merative MarketScan Research Databases, using *International Classification of Diseases, Ninth Revision, Clinical Modification* and *International Statistical Classification of Diseases, Tenth Revision, Clinical Modification* codes for cirrhosis or a clinical decompensating event (ascites, hepatorenal syndrome, hepatic encephalopathy, or variceal bleeding). Male and female patients were matched at a 1:1 ratio for age, etiologies of cirrhosis, geographic region, insurance type, specialty type, alcohol use disorder, obesity, baseline status of decompensation, and Charlson Comorbidity Index score. DC indicates decompensated cirrhosis; HCC, hepatocellular carcinoma; LT, liver transplant. ^a^Analysis was limited in patients with baseline-compensated cirrhosis.

After excluding ineligible patients, a total of 438 706 patients with cirrhosis (216 018 females [49.2%] and 222 688 males [50.8%]) were identified (mean [SD] age, 56.8 [15.4] years; females: 55.9 [16.4] years and males: 57.6 [14.3] years) (Figure 1). As shown in Table 1, before PSM, males were more likely than females to be referred to a GI/ID specialist (36.6% vs 26.9%) and had a numerically higher mean (SD) Charlson Comorbidity Index score (4.42 [2.47] vs 4.18 [2.37]) (SMD = 0.100). Males were more likely than females to have HBV (1.7% vs 0.7%), HCV (13.9% vs 7.1%), and ALD (26.3% vs 12.7%) but less likely to have MASLD (43.4% vs 53.6%), autoimmune liver diseases (3.1% vs 6.1%), and cryptogenic cirrhosis (9.8% vs 18.9%) (all SMD > 0.100 except SMD = 0.095 for comparison for HBV). PSM yielded 169 711 pairs of male and female patients with cirrhosis and balanced baseline characteristics (all SMD < 0.100) for inclusion in the analysis of adverse liver event incidence (Figure 1 and Table 1).

**Table 1.** Baseline Characteristics of Patients by Sex Before and After PSM^a^

Characteristic	Before PSM			After PSM^b^		
	Male (n = 222 688)	Female (n = 216 018)	SMD	Male (n = 169 711)	Female (n = 169 711)	SMD
Age, mean (SD), y	57.6 (14.3)	55.9 (16.4)	0.109	57.7 (15.2)	58.1 (15.9)	0.022
Region						
Northeast	42 113 (18.9)	39 940 (18.5)	0.016	32 423 (19.1)	31 129 (18.3)	0.031
North Central	50 973 (22.9)	50 038 (23.2)		39 592 (23.3)	40 308 (23.8)	
South	81 599 (36.6)	80 146 (37.1)		60 618 (35.7)	62 388 (36.8)	
West	35 188 (15.8)	33 472 (15.5)		26 859 (15.8)	26 295 (15.5)	
Unknown	12 815 (5.8)	12 422 (5.8)		10 219 (6.0)	9591 (5.7)	
Insurance type						
HMO	125 255 (56.2)	120 114 (55.6)	0.014	93 582 (55.1)	94 361 (55.6)	0.010
PPO	46 811 (21.0)	45 635 (21.1)		35 388 (20.9)	35 256 (20.8)	
Other	50 622 (22.7)	50 269 (23.3)		40 741 (24.0)	40 094 (23.6)	
Specialty type						
GI/ID	81 497 (36.6)	58 143 (26.9)	0.251	52 481 (30.9)	51 755 (30.5)	0.064
PCP	32 107 (14.4)	25 521 (11.8)		25 079 (14.8)	21 645 (12.8)	
Other	109 084 (49.0)	132 354 (61.3)		92 151 (54.3)	96 311 (56.8)	
Etiology						
HBV	3833 (1.7)	1486 (0.7)	0.095	2226 (1.3)	1486 (0.9)	0.027
HCV	30 976 (13.9)	15 412 (7.1)	0.222	15 508 (9.1)	15 415 (9.1)	0.004
ALD	58 557 (26.3)	27 541 (12.7)	0.347	29 471 (17.4)	27 340 (16.1)	0.044
MASLD	96 706 (43.4)	115 705 (53.6)	0.204	90 811 (53.5)	95 759 (56.4)	0.061
Autoimmune	6900 (3.1)	13 270 (6.1)	0.145	6823 (4.0)	6183 (3.6)	0.014
Cryptogenic	21 826 (9.8)	40 742 (18.9)	0.261	21 761 (12.8)	21 150 (12.5)	0.018
Other^c^	2205 (1.0)	1203 (0.6)	0.049	2136 (1.3)	1719 (1.0)	0.018
Baseline decompensation						
Compensated	132 710 (59.6)	143 674 (66.5)	0.144	106 139 (62.5)	110 936 (65.4)	0.059
Decompensated	89 978 (40.4)	72 344 (33.5)		63 572 (37.5)	58 775 (34.6)	
Type of decompensation						
Ascites	53 328 (23.9)	47 462 (22.0)	0.047	39 900 (23.5)	36 750 (21.7)	0.044
HRS	3891 (1.7)	2334 (1.1)	0.057	2724 (1.6)	2135 (1.3)	0.029
VH	31 906 (14.3)	18 288 (8.5)	0.185	19 580 (11.5)	17 069 (10.1)	0.048
HE	13 632 (6.1)	9250 (4.3)	0.083	9646 (5.7)	8155 (4.8)	0.039
Alcoholism	73 760 (33.1)	33 683 (15.6)	0.417	36 952 (21.8)	33 502 (19.7)	0.050
Obesity	30 680 (13.8)	35 855 (16.6)	0.079	26 513 (15.6)	25 402 (15.0)	0.018
Diabetes	57 481 (25.8)	46 561 (21.6)	0.100	45 060 (26.6)	42 717 (25.2)	0.032
Hypertension	108 057 (48.5)	92 635 (42.9)	0.113	83 882 (49.4)	80 574 (47.5)	0.039
Hyperlipidemia	69 573 (31.2)	55 608 (25.7)	0.122	54 413 (32.1)	52 136 (30.7)	0.029
CVD	31 371 (14.1)	21 669 (10.0)	0.125	23 425 (13.8)	21 307 (12.6)	0.037
CKD	25 383 (11.4)	18 478 (8.6)	0.095	19 392 (11.4)	17 705 (10.4)	0.032
CCI score, mean (SD)	4.42 (2.47)	4.18 (2.37)	0.100	4.49 (2.49)	4.43 (2.42)	0.022

Abbreviations: ALD, alcohol-associated liver disease; CCI, Charlson Comorbidity Index; CKD, chronic kidney disease; CVD, cardiovascular disease; GI/ID, gastroenterology or infectious disease; HBV, hepatitis B virus; HCV, hepatitis C virus; HE, hepatic encephalopathy; HMO, health maintenance organization; HRS, hepatorenal syndrome; MASLD, metabolic dysfunction-associated steatotic liver disease (formerly nonalcoholic fatty liver disease [NAFLD]); PCP, primary care physician; PPO, preferred provider organization; PSM, propensity score matching; SMD, standardized mean difference; VH, variceal hemorrhage.

^a^ Data are presented as No. (%) of patients unless otherwise indicated.

^b^ Matched for age, etiologies of cirrhosis, geographic region, insurance type, specialty type, alcoholism, obesity, decompensation, and CCI score.

^c^ Included autoimmune hepatitis, primary biliary cholangitis, primary sclerosing cholangitis, Wilson diseases, hemochromatosis, and α-1 antitrypsin deficiency.

### Incidence and Risk of Adverse Liver Events Between Matched Male and Female Patients

#### Overall Cohort

**Figure 2.**
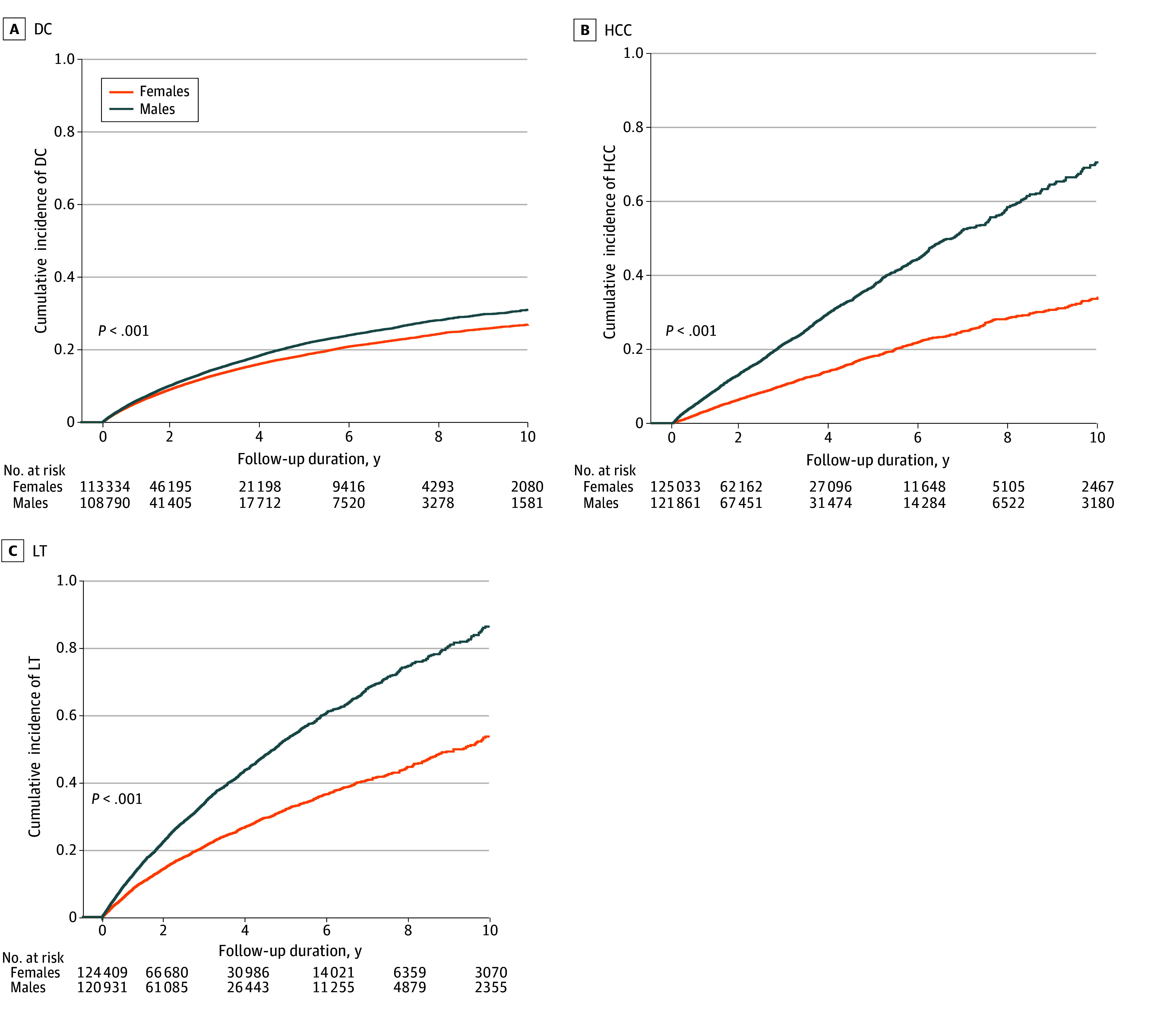
Cumulative Incidence of Decompensated Cirrhosis (DC), Hepatocellular Carcinoma (HCC), and Liver Transplant (LT) in Patients With Cirrhosis, by Sex Survival probabilities were estimated by the Kaplan-Meier approach and compared by the log-rank test.

Over a total follow-up of 258 178.2 person-years (PYs) for females and 228 004.2 PYs for males, DC was identified in 113 334 females (265 766.1 PYs), HCC in 125 033 females (377 919.8 PYs), and LT in 124 409 females (373 369.7 PYs); among males, 108 790 (236 352.3 PYs) were identified with DC, 121 861 (344 422.4 PYs) with HCC, and 120 931 (338 305.7 PYs) with LT. Males had significantly higher incidence rates per 1000 PYs than did females for DC (65.77 [95% CI, 64.74-66.81] vs 55.35 [95% CI, 54.46-56.25]; *P* < .001), HCC (6.98 [95% CI, 6.71-7.27] vs 3.35 [95% CI, 3.17-3.54]; *P* < .001), and LT (10.23 [95% CI, 9.89-10.58] vs 6.27 [95% CI, 6.01-6.52]; *P* < .001) (Table 2). Males compared with females also had a higher 10-year cumulative incidence of DC (34.4% vs 29.9%), HCC (7.1% vs 3.4%), and LT (9.6% vs 5.4%) (all *P* < .001) (Figure 2). In a Cox proportional hazards regression analysis, males had a 16% higher risk of DC (HR, 1.16 [95% CI, 1.14-1.19]; *P* < .001), 110% higher risk of HCC (HR, 2.10 [95% CI, 1.96-2.25]; *P* < .001), and 63% higher risk of LT (HR, 1.63 [95% CI, 1.54-1.71]; *P* < .001) compared with females (Table 2). The absolute risk differences between females and males for DC, HCC and LT were −0.0104 (95% CI, −0.0118 to −0.0091), −0.0036 (95% CI, −0.0040 to −0.0033) and −0.0040 (95% CI, −0.0044 to −0.0036), respectively (all *P* < .001). Sensitivity analysis using inverse probability of treatment weighting also yielded consistent findings (eTable 2 in Supplement 1). Finally, the E-values were 1.59 for DC, 3.62 for HCC, and 2.64 for LT, suggesting that an unmeasured confounder would need to have a significant association with both exposure and outcome to fully explain away the observed associations.

**Table 2.** Association Between Sex and Adverse Liver Events in the Total Cohort^a^

Event	Patients, No.	Person-years	Events, No.	Incidence per 1000 person-years of event (95% CI)	*P* value	HR (95% CI)	Absolute risk difference (95% CI)^b^	*P* value
**DC**								
Male	108 790	236 352.3	15 545	65.77 (64.74 to 66.81)	<.001	1.16 (1.14 to 1.19)	−0.0104 (−0.0118 to −0.0091)	<.001
Female	113 334	265 766.1	14 710	55.35 (54.46 to 56.25)		1 [Reference]		
**HCC**								
Male	121 861	344 422.4	2405	6.98 (6.71 to 7.27)	<.001	2.10 (1.96 to 2.25)	−0.0036 (−0.0040 to −0.0033)	<.001
Female	125 033	377 919.8	1266	3.35 (3.17 to 3.54)		1 [Reference]		
**LT**								
Male	120 931	338 305.7	3461	10.23 (9.89 to 10.58)	<.001	1.63 (1.54 to 1.71)	−0.0040 (−0.0044 to −0.0036)	<.001
Female	124 409	373 369.7	2339	6.27 (6.01 to 6.52)		1 [Reference]		

Abbreviations: DC, decompensated cirrhosis; HCC, hepatocellular carcinoma; HR, hazard ratio; LT, liver transplant.

^a^ Statistical analysis was performed by a Cox proportional hazards regression model.

^b^ Calculated by risk in females minus risk in males.

#### Subgroup Analyses

Subgroup analyses stratified by major liver disease etiologies (HBV, HCV, ALD, and MASLD) were performed. Overall, males had higher incidence rates per 1000 PYs of DC, HCC, and LT than females in each subgroup except for the group of patients with HBV, for which males only had higher per-1000 PYs incidence of HCC than females (13.76 [95% CI, 10.97-17.03] vs 8.63 [95% CI, 6.04-11.95]; *P* = .02). Specifically, males with ALD had the highest incidence rates per 1000 PYs of DC (144.30 [95% CI, 139.80-148.90]; *P* < .001); males with HCV had the highest incidence rates of DC (138.20 [95% CI, 133.80-142.80]; *P* < .001); while females with MASLD had the lowest incidence rates of DC (32.32 [95% CI, 31.44-33.22]; *P* < .001), HCC (1.17 [95% CI, 1.03-1.33]; *P* < .001), as well as LT (2.29 [95% CI, 2.09-2.50]; *P* < .001) (eTable 3 in Supplement 1).

**Figure 3.**
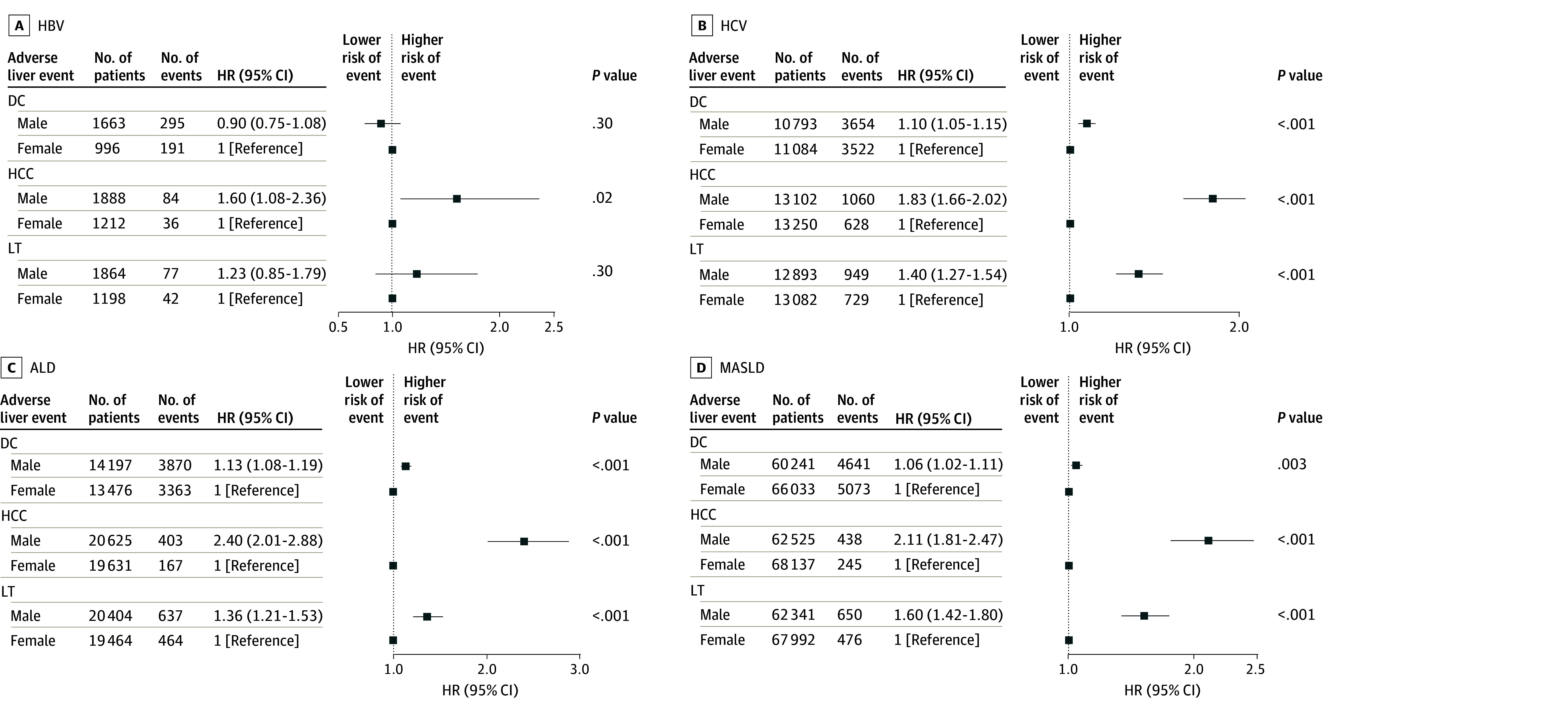
Association Between Sex and Adverse Liver Events in Patients with Hepatitis B Virus (HBV), Hepatitis C Virus (HCV), Alcohol-Associated Liver Disease (ALD), and Metabolic Dysfunction-Associated Steatotic Liver Disease (MASLD) Cirrhosis Etiologies Hazard ratios (HRs) and 95% CIs for decompensated cirrhosis (DC), hepatocellular carcinoma (HCC), and liver transplant (LT) were estimated by a Cox proportional hazards regression model.

In a Cox proportional hazards regression analysis, male patients compared with female patients with ALD-associated cirrhosis had the highest risk of developing adverse liver events, with 13% higher risk of DC (HR, 1.13 [95% CI, 1.08-1.19]; *P* < .001), 140% higher risk of HCC (HR, 2.40 [95% CI, 2.01-2.88]; *P* < .001), and 36% higher risk of LT (HR, 1.36 [95% CI, 1.21-1.53]; *P* < .001), followed by MASLD and HCV, while there were no significant sex differences among patients with HBV, except for those with HCC, among whom male patients had higher risk (HR, 1.60 [95% CI, 1.08-2.36]; *P* = .02) (Figure 3). Among patients with viral hepatitis and concurrent MASLD or ALD, we found a significant association between male sex and HCC in the HBV without MASLD group (HR, 3.31 [95% CI, 1.13-9.71]) and significant associations between male sex and all adverse liver events (DC, HCC, and LT) for all HCV groups except for the combined HCV with MASLD and HCV with ALD etiology groups and the DC event (eTables 4 and 5 in Supplement 1).

In subgroup analyses stratified by age and the presence of baseline DC, compared with female patients, male patients consistently had higher incidence and risk of adverse liver events in all subgroups regardless of age and regardless of the presence of baseline DC (eFigures 1 and 2 and eTables 6 and 7 in Supplement 1). In subgroup analysis by time period of cirrhosis diagnosis, the differences in the risk of adverse liver events between male and female patients were more prominent in the cohort with cirrhosis diagnosed before or in 2014 compared with those with a cirrhosis diagnosis after 2014 (eTable 8 in Supplement 1). In subgroup analyses stratified by the indications for LT (HCC vs non-HCC), males were more likely to undergo LT for both HCC and non-HCC indications than females, but the difference between males and females was more remarkable with HCC indication (HR, 2.42 [95% CI, 2.14-2.75) than with non-HCC indication (HR, 1.48 [95% CI, 1.40-1.57]) (both *P* < .001) (eTable 9 in Supplement 1).

## Discussion

In this large-scale study investigating sex disparities in the risk of adverse liver events among a nationwide cohort of US patients with private health insurance, we observed a similar number of female and male patients with cirrhosis. Yet, in matched analyses of females and males with cirrhosis and balanced baseline characteristics, males had over a 100% higher risk of HCC, a 63% higher risk of LT, and a 16% higher risk of DC than females. We also found that the sex difference in the risk of adverse liver events was more pronounced in nonviral cirrhosis compared with viral cirrhosis. Considering the shifting etiologies of cirrhosis from viral to nonviral in recent years, future prevention and surveillance strategies for cirrhosis-related complications should incorporate these sex differences.

Consistent with our findings, a National Inpatient Sample-based study of hospitalized patients with cirrhosis across the US reported fewer hepatic decompensation events in females than in males.^8^ Another study using an electronic health record database from Chicago, Illinois, also found that females were less likely than males to develop portal hypertension-related complications.^10^ However, both studies analyzed only prevalent data and were limited to hospitalized or tertiary care settings. Our study expanded these findings to a broader population, encompassing inpatient and outpatient encounters from both tertiary and community clinical centers and from both specialist and primary care settings. Additionally, our study provided new evidence showing a 16% increased risk of developing DC in male patients with cirrhosis during a longitudinal 10-year follow-up.

We found even more pronounced sex differences in analyses of HCC outcome, in which males had a 110% higher risk of HCC than females in our study; these findings are in line with results from prior studies.^10,15,16^ Sex hormones, particularly androgens, which predominate in males, and estrogens in females, are believed to play a major biological role in the sex-based disparity in HCC risk.^17^ Additionally, compared with females, males are more likely to consume alcohol, smoke cigarettes, and have type 2 diabetes and visceral obesity, all of which are associated with an increased risk of HCC.^18,19^ Furthermore, men generally demonstrate lower adherence to surveillance programs compared with women, which may contribute to delayed diagnoses and poorer survival outcomes for HCC.^20^ Together with prior studies, our findings support the current HCC surveillance strategies in patients with cirrhosis, but more intensive strategies may be warranted for HCC surveillance in male patients with cirrhosis.

In our study, female patients with cirrhosis had a lower incidence rate and likelihood of LT than male patients. These findings are consistent with data showing that more males than females are on the waiting lists for both the United Network for Organ Sharing and the Eurotransplant database.^21^ On one hand, females had fewer DC and HCC cases than males, both of which are major indications for transplant in patients with cirrhosis. On the other hand, even with the same severity, females appeared to be less likely to receive a LT than males,^22,23^ and there may be several reasons for this discrepancy. First, some may suggest that it could be related to serum creatinine, a component of the Model for End-Stage Liver Disease (MELD) score, which among females underestimates the severity of kidney dysfunction, resulting in lower transplant priority.^24^ Second, females generally have smaller body sizes than males, which reduces the likelihood of suitable organ matching.^25^ Notably, we observed a larger sex-based difference in the likelihood of LT for HCC compared with non-HCC. This may be related to a larger difference in HCC risk than DC risk between males and females. Additionally, female patients with HCC were more likely to undergo resection at earlier disease stages than males, thus potentially decreasing their need for LT.^26^

Although all of these suggestions need further research, data from the United Network for Organ Sharing^27^ system recently reported that the largest reason for inequity in LT was due to the donation service area, in which a combination of factors come into play including geographic variation in inherent organ supply relative to demand, transplant hospital practice variations, availability of organs, and allocation policy priority for local candidates. In addition, systemic biases persist that can contribute to inequities in LT,^28^ with reports that Black and Hispanic patients have lower rates of LT waitlisting compared with their White counterparts.^28^ Limited insurance coverage and lower socioeconomic status in racial and ethnic minority populations may further hinder referral and waitlisting for transplant.^28^ Moreover, undocumented immigrants often face legal and policy barriers that restrict access to transplant evaluation and care.^29^

An interesting finding of this study is the sex differences in the risk of adverse liver events among those with viral hepatitis-related cirrhosis. In HBV cirrhosis, males had a higher risk of HCC but not of DC and LT, while males with HCV-related cirrhosis had a higher risk of all 3 studied outcomes. The less pronounced sex disparity in HBV-related outcomes compared with HCV may be partly due to the smaller sample size; however, we suggest that it may also be due to a higher use of antiviral medications for those with HBV compared with HCV.^30^ Although, direct acting antiviral medications have now been available for over a decade, their uptake has taken time to increase,^31^ so there may be lag in these respective outcomes requiring future investigations.

Despite females’ higher susceptibility to alcohol-induced liver injury due to lower gastric and hepatic alcohol dehydrogenase activity, smaller alcohol distribution volumes, and the differential influence of alcohol on sex hormone expression,^32,33^ male patients with ALD cirrhosis exhibited higher risk of adverse liver events than females. This paradox may be explained because men typically consume higher amounts of alcohol and are more likely to engage in heavy drinking.^34^ It may also be due to behavioral differences, as females are more likely to abstain from alcohol due to health concerns, family responsibilities, or social expectations.^35^ Moreover, females are less likely to relapse after abstinence and more likely to seek supportive therapies, such as counseling or group interventions.^36^ Emerging evidence also suggests biological mechanisms underlying the observed sex differences in the progression of ALD-related cirrhosis. For example, animal studies have demonstrated a sexually dimorphic response to alcohol, with alcohol specifically activating lysine demethylase 5B in male mice, promoting hepatocyte dedifferentiation and subsequent tumor development, an outcome not observed in female mice.^37^ However, it should be acknowledged that sex differences in ALD-related adverse events may also be influenced by differences in alcohol consumption,^34^ the presence of secondary liver disease etiologies such as viral hepatitis, and other unmeasured confounders not reported in the database.

Sex differences also were associated with various stages of MASLD progression.^38^ MASLD prevalence is generally lower in females than males throughout the lifespan^39^ due to the protection from estrogen.^40,41^ On the other hand, males are more likely to develop metabolic dysfunction-associated steatohepatitis and fibrosis up to a certain age due to the effects of testosterone on the liver and the presence of visceral obesity and insulin resistance.^42,43^ However, after menopause when estrogen protection is lost, disease prevalence and progression in females are shown to become more similar to males.^6,44^ These findings were also highlighted in our study, in which we observed that females, who had a mean age of 56 years, were more likely to have MASLD cirrhosis, but males were still at significantly higher risks of DC, HCC, and LT compared with females. The higher risk may be due to males with MASLD being more likely to have concurrent alcohol use, which can exacerbate mortality risks, even with moderate consumption.^45^ The sex disparity in MASLD-related cirrhosis may be further explained by the higher expression of liver formyl peptide receptor 2 in female mice.^46^ As an estrogen-regulated modulator of inflammatory responses, formyl peptide receptor 2 confers greater protection against MASLD progression in females. Regarding MASLD-HCC pathogenesis, increased interleukin-6 production from Kupffer cells has been observed in males, while estrogen in females suppresses interleukin-6 secretion, leading to attenuated chronic inflammation and reduced hepatocarcinogenesis risk in females.^47^ Meanwhile, the molecular subgroup of HCA harboring mutations of β-catenin in exon 3 was more frequently observed in males, which is significantly associated with androgen exposure and HCC development.^47^

Another interesting finding of the current study is that cirrhosis diagnoses were more likely to be made by GI/ID specialists in males than females, even though the proportion of DC between males and females at index diagnosis was similar, so the presence of DC was unlikely the factor that may have led more males to a GI/ID specialist. Instead, it may be related to sex differences in cirrhosis etiologies. Males are more likely to have viral hepatitis or ALD, conditions more commonly managed by specialists, whereas females more frequently had MASLD or cryptogenic cirrhosis and were more likely to consult other specialties, which may have been due to their extrahepatic comorbidities.

### Strengths and Limitations

A strength of our study is its large, nationally representative, and diverse population. The inclusion of inpatient, outpatient, and outpatient pharmacy encounter data and longitudinal follow-up at both primary and tertiary care settings further enhanced the robustness of our findings. In addition, we carefully balanced the baseline characteristics of males and females to minimize potential confounding associations.

However, the study had several limitations. First, the diagnoses of cirrhosis, liver disease etiologies, and adverse liver events relied on *ICD-9-CM* and *ICD-10-CM* codes, posing undercoding or miscoding risks, but such inherent limitations of claims databases are likely to affect males and females similarly; to mitigate this, we only included patients with at least 1 inpatient or 2 outpatient diagnoses of cirrhosis or its complications. Second, the lack of laboratory data prevented the calculation of the MELD with sodium levels (MELD-Na) and Child-Pugh scores for disease severity comparison. However, we used validated *ICD-9-CM* and *ICD-10-CM* codes for identifying cirrhosis complications and estimating decompensation as an alternative severity marker. Even so, potential residuals confounding the outcome could still remain, although the high E-values suggest that the potential confounders would have had to be significantly associated with both our variables and outcomes to fully explain away the observed outcomes. Third, we were unable to perform competing risk analyses due to the lack of mortality data in this database. However, as male patients with cirrhosis have been reported to experience higher mortality rates than females,^9^ the absence of mortality data may have led to an underestimation of the sex-based differences in the risk of liver-related adverse events but would not change the overall direction of our conclusions. Lastly, the database includes only privately insured individuals, which may introduce selection bias and limit the generalizability of our findings, thus requiring validation in uninsured populations that are more likely to have a lower socioeconomic status and may have more severe disease as a result.

## Conclusions

The findings of this cohort study advance understanding of sex differences in clinical outcomes of cirrhosis. The key finding is that male patients have higher risk of adverse liver events than females, in particular, the risk of HCC. Such sex differences were larger in nonviral cirrhosis compared with viral cirrhosis. As MASLD and ALD are leading causes of cirrhosis in high-income countries and are projected to rise globally, future cirrhosis management strategies, in particular HCC surveillance, should be sex-based. Our findings support incorporating sex as a variable in risk stratification for cirrhosis-related complications. Given the higher risk of HCC in males with cirrhosis, targeted patient and practitioner education is needed to help improve the current low rates of adherence to HCC surveillance, as only about 10% of privately insured US patients with cirrhosis overall undergo abdominal imaging every 6 to 12 months.^48^ Prospective studies incorporating laboratory measures (eg, MELD-Na) are also warranted to validate our conclusions. In addition, further research is needed to identify modifiable factors underlying these sex-based differences, such as differences in medication adherence or lifestyle factors, for potential interventions.

## Notes

Supplement 1. **eTable 1.** *ICD-9-CM/ICD-10-CM* Codes for the Study **eTable 2.** Association Between Sex and Adverse Liver Events in Overall Cohort by IPTW **eTable 3.** Association Between Sex and Adverse Liver Events in Patients With Cirrhosis and HBV (A), HCV (B), ALD (C), and MASLD (D) **eTable 4.** Association Between Sex and Adverse Liver Events in Patients With Cirrhosis and HBV With MALSD (A), HBV Without MASLD (B), HBV With ALD (C), and HBV Without ALD (D) **eTable 5.** Association Between Sex and Adverse Liver Events in Patients With Cirrhosis and HCV With MALSD (A), HCV Without MASLD (B), HCV With ALD (C), and HCV Without ALD (D) **eTable 6.** Association Between Sex and Adverse Liver Events in Subgroup by Age **eTable 7.** Association Between Sex and Adverse Liver Events in Subgroup by Presence of Hepatic Decompensation **eTable 8.** Association Between Sex and Adverse Liver Events in Subgroup by the Time Periods of Cirrhosis Diagnosis **eTable 9.** Association Between Sex and Liver Transplantation in Subgroup by HCC and Non-HCC **eFigure 1**. Cumulative Incidence of DC (A), HCC (B), and LT (C) in Patients With Cirrhosis, by Sex and Age **eFigure 2**. Cumulative Incidence of HCC (A) and LT (B) in Patients With Cirrhosis, by Sex and Baseline Decompensation

Supplement 2.
Data Sharing Statement
