# Supplementary material for: Sex Differences in Risk of Adverse Liver Events in Patients With Cirrhosis
Source: JAMA Netw Open. 2025 Jul 28;8(7):e2523674. doi: 10.1001/jamanetworkopen.2025.23674 (PMC12305389; doi:10.1001/jamanetworkopen.2025.23674)
Supplement: Supplement 1. — eTable 1. ICD-9-CM/ICD-10-CM Codes for the Study eTable 2. Association Between Sex and Adverse Liver Events in Overall Cohort by IPTW eTable 3. Association Between Sex and Adverse Liver Events in Patients With Cirrhosis and HBV (A), HCV (B), ALD (C), and MASLD (D) eTable 4. Association Between Sex and Adverse Liver Events in Patients With Cirrhosis and HBV With MALSD (A), HBV Without MASLD (B), HBV With ALD (C), and HBV Without ALD (D) eTable 5. Association Between Sex and Adverse Liver Events in Patients With Cirrhosis and HCV With MALSD (A), HCV Without MASLD (B), HCV With ALD (C), and HCV Without ALD (D) eTable 6. Association Between Sex and Adverse Liver Events in Subgroup by Age eTable 7. Association Between Sex and Adverse Liver Events in Subgroup by Presence of Hepatic Decompensation eTable 8. Association Between Sex and Adverse Liver Events in Subgroup by the Time Periods of Cirrhosis Diagnosis eTable 9. Association Between Sex and Liver Transplantation in Subgroup by HCC and Non-HCC eFigure 1. Cumulative Incidence of DC (A), HCC (B), and LT (C) in Patients With Cirrhosis, by Sex and Age eFigure 2. Cumulative Incidence of HCC (A) and LT (B) in Patients With Cirrhosis, by Sex and Baseline Decompensation [file jamanetwopen-e2523674-s001.pdf]

Supplemental Online Content

Shi Y, Zhang X, Wong T, et al. Sex differences in risk of adverse liver events in patients with cirrhosis. *JAMA Netw Open*. 2025;8(7):e2523674. doi:10.1001/jamanetworkopen.2025.23674

eTable 1. *ICD-9-CM/ICD-10-CM* Codes for the Study
eTable 2. Association Between Sex and Adverse Liver Events in Overall Cohort by IPTW
eTable 3. Association Between Sex and Adverse Liver Events in Patients With Cirrhosis and HBV (A), HCV (B), ALD (C), and MASLD (D)
eTable 4. Association Between Sex and Adverse Liver Events in Patients With Cirrhosis and HBV With MALSD (A), HBV Without MASLD (B), HBV With ALD (C), and HBV Without ALD (D)
eTable 5. Association Between Sex and Adverse Liver Events in Patients With Cirrhosis and HCV With MALSD (A), HCV Without MASLD (B), HCV With ALD (C), and HCV Without ALD (D)
eTable 6. Association Between Sex and Adverse Liver Events in Subgroup by Age
eTable 7. Association Between Sex and Adverse Liver Events in Subgroup by Presence of Hepatic Decompensation
eTable 8. Association Between Sex and Adverse Liver Events in Subgroup by the Time Periods of Cirrhosis Diagnosis
eTable 9. Association Between Sex and Liver Transplantation in Subgroup by HCC and Non-HCC
eFigure 1. Cumulative Incidence of DC (A), HCC (B), and LT (C) in Patients With Cirrhosis, by Sex and Age
eFigure 2. Cumulative Incidence of HCC (A) and LT (B) in Patients With Cirrhosis, by Sex and Baseline Decompensation

This supplemental material has been provided by the authors to give readers additional information about their work.

© 2025 Shi Y et al. *JAMA Network Open*.

eTable 1. *ICD-9-CM/ICD-10-CM* Codes for the Study

Disease	*ICD-9-CM/ICD-10-CM* diagnosis codes/procedure codes
Liver cirrhosis	571.2, 571.5, 572.3, 456.1, 456.21;<br>K70.30, K74.60, K74.69, K76.6, I85.00, I85.10
Hepatic decompensation	
Ascites	789.5, 789.59; R18. 8, K70.31
Variceal bleeding	456.0, 456.2, 456.8; I85.01, I85.11, I86.4
Hepatic encephalopathy	572.2; K76.82, K72.91, K72.01, K72.11
Hepatorenal syndrome	572.4; K76.7
Hepatocellular carcinoma	155.0; C22.0, C22.8
Liver transplant	V42.7, 996.82; 50.51*, 50.59*; Z94.4, T86.43, T86.40; 0FY00Z0*, 0FY00Z1*, 0FY00Z2*
HBV	070.20-070.23, 070.30-070.33, V02.61; B16.0, B16.1, B16.9, B18.0, B18.1, B19.1, B19.10, B19.11
HCV	070.41, 070.44, 070.51, 070.54, 070.70, 070.71, V02.62; B17.1, B19.2, B17.11, B18.2, B17.10, B19.20, B19.21, Z22. 52
Significant alcohol use	291, 291.0-291.5, 291.81, 291.82, 291.89, 291.9, 303, 303.0-303.03, 303.9, 303.90-303.93, 305, 305.00-305.03, 357.5, 425.5, 535.3, 535.30, 535.31, 571.0, 571.1 571.2, 571.3, 655.4, 760.71, V11.3, E860.0; F10, F10.1, F10.2, F10.9, G31.2, G72.1, I42.6, K29.20, K29.21, K85.2, K86.0, Z71.4, G62.1, E24.4, K70.0-K70.4, K70.9, Q86.0, P04.3, O35.4, O99.31, T51
MASLD	571.8, 571.9; K76.0, K75.81
Autoimmune hepatitis	571.42; K75.4
Primary biliary cirrhosis	571.6; K74.3
Primary sclerosing cholangitis	2576.1; K83. 01
Wilson disease	275.1; E83.01
Hemochromatosis	275.01, 275.02, 275.03; E83.11, E83.110, E83.111, E83.118, E83.119
Alpha-1-antitrypsin deficiency	273.4; E88.01
Obesity	278.00, 278.01, 278.03, V85.30-V85.39, V85.41-V85.45; E66.0, E66.01, E66.09, E66.1, E66.2, E66.8, E66.9; Z68.30-Z68.39; Z68.41-Z68.45
Diabetes	250.0X-250.9X; E10.X-E11.X, E13.X
Hypertension	401, 401.0, 401.1, 401.9; I10
Hyperlipidemia	272.0-272.4; E78.00, E78.1- E78.4, E78.49, E78.5
Cardiovascular diseases	398.91, 402,01, 402.11, 402.91, 404.01, 404.03, 404.11, 404.13, 404.91, 404.93, 425.4-425.9, 428.0, 428.1, 428.20-428.23, 428.30-428.33, 428.40-428.43, 428.9;<br>I20. 9, I21.0-I21.9, I21.A1, I21.A9; I25, I25.1, I25.10, I25.11X, I25.2, I25.5, I25.84, I25.9; I42.0-I42.9; I43; I50.1-I50.4, I50.8, I50.9
Chronic kidney disease	585.1-585.6, 585.9; 403.00, 403.01, 403.10, 403.11, 403.90, 403.91; 404.00-404.03, 404.10-404.13, 404.90-404.93; V42.0, 996.81, 55.69*;<br>N18.1-N18.6, N18.9; I12.0, I12.9; E11.22, E10.22; T86.10, Z94.0, Z48.22

*represent procedural codes. Abbreviation: HBV, hepatitis B virus; HCV, hepatitis C virus; MASLD, metabolic dysfunction-associated steatotic liver disease.

© 2025 Shi Y et al. *JAMA Network Open*.

eTable 2. Association Between Sex and Adverse Liver Events in Overall Cohort by IPTW

Events	No. of patients	No. of events	HR (95% CI)	*P* value
**DC**				
Male	109832	17237	1.06 (1.04, 1.09)	<0.001
Female	116111	11614	Ref	
**HCC**				
Male	162047	4348	1.97 (1.85, 2.10)	< 0.001
Female	161324	1381	Ref	
**LT**				
Male	160714	5567	1.52 (1.44, 1.59)	<0.001
Female	160580	2770	Ref	

Statistical analysis was performed by a Cox-proportional hazard model. Patients in male and female groups were matched by IPTW for age, etiologies of cirrhosis, race/ethnicity, geographic region, insurance type, specialty type, alcohol use disorder, BMI or obesity, baseline status of decompensation (defined as the presence of ascites, HRS, hepatic encephalopathy, or variceal bleeding), and CCI. All SMD< 0.1. Abbreviation: DC, decompensated cirrhosis; HCC, hepatocellular carcinoma; LT, liver transplantation; HR, hazard ratio; CI, confidence interval

© 2025 Shi Y et al. *JAMA Network Open*.

eTable 3. Association Between Sex and Adverse Liver Events in Patients With Cirrhosis and HBV (A), HCV (B), ALD (C), and MASLD (D)

	No. of patients	Person-years	No. of events	Incidence of events[#]	HR (95% CI)
**A. HBV**					
**DC**					
Male	1663	4740.2	295	62.23 (55.33, 69.76)	0.90 (0.75, 1.08)
Female	996	2802.3	191	68.16 (58.83, 78.54)	Ref
**HCC**					
Male	1888	6106	84	13.76 (10.97, 17.03)*	1.60 (1.08, 2.36)*
Female	1212	4171.7	36	8.63 (6.04, 11.95)	Ref
**LT**					
Male	1864	6018.9	77	12.79 (10.10, 15.99)	1.23 (0.85, 1.79)
Female	1198	4078.7	42	10.30 (7.42, 13.92)	Ref
**B. HCV**					
**DC**					
Male	10793	26432.6	3654	138.2 (133.8, 142.8)***	1.10 (1.05, 1.15)***
Female	11084	28316.4	3522	124.4 (120.3, 128.6)	Ref
**HCC**					
Male	13102	38967.7	1060	27.2 (25.59, 28.89)***	1.83 (1.66, 2.02)***
Female	13250	42205.7	628	14.88 (13.74, 16.09)	Ref
**LT**					
Male	12893	38191.5	949	24.85 (23.29, 26.48)***	1.40 (1.27, 1.54)***
Female	13082	41233.2	729	17.68 (16.42, 19.01)	Ref
**C. ALD**					
**DC**					
Male	14197	26819.1	3870	144.3 (139.8, 148.9)***	1.13 (1.08, 1.19)***
Female	13476	26904.2	3363	125.0 (120.8, 129.3)	Ref
**HCC**					
Male	20625	55148	403	7.31 (6.61, 8.06)***	2.40 (2.01, 2.88)***
Female	19631	54601.7	167	3.06 (2.61, 3.56)	Ref
**LT**					
Male	20404	53940.2	637	11.81 (10.91, 12.76)***	1.36 (1.21, 1.53)***
Female	19464	53549.4	464	8.67 (7.89, 9.49)	Ref
**D. MASLD**					
**DC**					
Male	60241	132014.1	4641	35.16 (34.15, 36.18)***	1.06 (1.02, 1.11)**
Female	66033	156968.7	5073	32.32 (31.44, 33.22)	Ref
**HCC**					
Male	62525	178241.8	438	2.46 (2.23, 2.70)***	2.11 (1.81, 2.47)***
Female	68137	209117.7	245	1.17 (1.03, 1.33)	Ref

© 2025 Shi Y et al. *JAMA Network Open*.

**LT**					
Male	62341	176884.2	650	3.68 (3.40, 3.97)***	1.60 (1.42, 1.80)***
Female	67992	208096.4	476	2.29 (2.09, 2.50)	Ref

Statistical analysis was performed by a Cox-proportional hazard model.

Abbreviation: HBV, hepatitis B virus; HCV, hepatitis C virus; ALD, alcohol-associated liver diseases; MASLD, metabolic dysfunction-associated steatotic liver disease; DC, decompensated cirrhosis; HCC, hepatocellular carcinoma; LT, liver transplantation; HR, hazard ratio; CI, confidence interval.

#represents per 1000 person-years of events.

For comparison with females: *P<0.05, **P<0.01, ***P<0.001.

© 2025 Shi Y et al. *JAMA Network Open*.

eTable 4. Association Between Sex and Adverse Liver Events in Patients With Cirrhosis and HBV With MALSD (A), HBV Without MASLD (B), HBV With ALD (C), and HBV Without ALD (D)

	No. of patients	No. of events	HR (95% CI)	P value
**A. HBV with MASLD**				
**DC**				
Male	1114	247	0.95 (0.78, 1.17)	0.65
Female	696	159	Ref	
**HCC**				
Male	1329	64	1.39 (0.91, 2.12)	0.13
Female	892	32	Ref	
**LT**				
Male	1307	67	1.16 (0.78, 1.71)	0.47
Female	881	40	Ref	
**B. HBV without MASLD**				
**DC**				
Male	549	48	0.75 (0.48, 1.15)	0.19
Female	300	35	Ref	
**HCC**				
Male	559	20	3.31 (1.13, 9.71)	0.03
Female	320	4	Ref	
**LT**				
Male	557	10	2.98 (0.65, 13.6)	0.16
Female	317	2	Ref	
**C. HBV with ALD**				
**DC**				
Male	145	57	0.89 (0.60, 1.32)	0.56
Female	101	45	Ref	
**HCC**				
Male	233	12	2.74 (0.88, 8.49)	0.08
Female	181	4	Ref	
**LT**				
Male	227	24	1.18 (0.63, 2.19)	0.61
Female	177	17	Ref	
**D. HBV without ALD**				
**DC**				
Male	1518	238	0.94 (0.76, 1.15)	0.54
Female	895	146	Ref	
**HCC**				
Male	1655	72	1.48 (0.97, 2.24)	0.07
Female	1031	32	Ref	

© 2025 Shi Y et al. *JAMA Network Open*.

**LT**				
Male	1637	51	1.39 (0.86, 2.23)	0.18
Female	1021	25	Ref	

Statistical analysis was performed by a Cox-proportional hazard model.

Abbreviation: HBV, hepatitis B virus; HCV, hepatitis C virus; ALD, alcohol-associated liver diseases; MASLD, metabolic dysfunction-associated steatotic liver disease; DC, decompensated cirrhosis; HCC, hepatocellular carcinoma; LT, liver transplantation; HR, hazard ratio; CI, confidence interval.

© 2025 Shi Y et al. *JAMA Network Open*.

eTable 5. Association Between Sex and Adverse Liver Events in Patients With Cirrhosis and HCV With MALSD (A), HCV Without MASLD (B), HCV With ALD (C), and HCV Without ALD (D)

	No. of patients	No. of events	HR (95% CI)	*P value*
**A. HCV with MASLD**				
**DC**				
Male	8974	3117	1.05 (0.99, 1.10)	0.08
Female	8644	2949	Ref	
**HCC**				
Male	10979	959	1.76 (1.59, 1.96)	<0.001
Female	10445	559	Ref	
**LT**				
Male	10804	874	1.32 (1.19, 1.46)	<0.001
Female	10309	672	Ref	
**B. HCV without MASLD**				
**DC**				
Male	1819	537	1.39 (1.23, 1.56)	<0.001
Female	2440	573	Ref	
**HCC**				
Male	2123	101	2.24 (1.65, 3.04)	<0.001
Female	2805	69	Ref	
**LT**				
Male	2089	75	1.95 (1.38, 2.76)	<0.001
Female	2773	57	Ref	
**C. HCV with ALD**				
**DC**				
Male	2207	1164	1.05 (0.97, 1.15)	0.22
Female	1996	1039	Ref	
**HCC**				
Male	3255	359	1.87 (1.57, 2.23)	<0.001
Female	2976	193	Ref	
**LT**				
Male	3191	360	1.30 (1.11, 1.52)	0.001
Female	2915	272	Ref	
**D. HCV without ALD**				
**DC**				
Male	8586	2490	1.08 (1.03, 1.15)	0.005
Female	9088	2483	Ref	
**HCC**				
Male	9847	701	1.79 (1.59, 2.02)	<0.001
Female	10274	435	Ref	

© 2025 Shi Y et al. *JAMA Network Open*.

**LT**				
Male	9702	589	1.42 (1.26, 1.61)	<0.001
Female	10167	457	Ref	

Statistical analysis was performed by a Cox-proportional hazard model.

Abbreviation: HBV, hepatitis B virus; HCV, hepatitis C virus; ALD, alcohol-associated liver diseases; MASLD, metabolic dysfunction-associated steatotic liver disease; DC, decompensated cirrhosis; HCC, hepatocellular carcinoma; LT, liver transplantation; HR, hazard ratio; CI, confidence interval.

© 2025 Shi Y et al. *JAMA Network Open*.

eTable 6. Association Between Sex and Adverse Liver Events in Subgroup by Age

	Age ≤57				Age >57			
	**No. of patients**	**No. of events**	**HR (95% CI)**	***P* value**	**No. of patients**	**No. of events**	**HR (95% CI)**	***P* value**
**DC**								
Male	56225	7752	1.20 (1.17, 1.24)	<0.001	52565	7793	1.13 (1.10, 1.17)	<0.001
Female	57371	6938	Ref		55963	7772	Ref	
**HCC**								
Male	66308	1103	2.12 (1.92, 2.35)	<0.001	57138	1302	2.08 (1.90, 2.28)	<0.001
Female	64723	580	Ref		58725	686	Ref	
**LT**								
Male	64122	2271	1.61 (1.51, 1.72)	<0.001	56809	1190	1.66 (1.51, 1.81)	<0.001
Female	65912	1560	Ref		58497	779	Ref	

Statistical analysis was performed by a Cox-proportional hazard model.
Abbreviation: DC, decompensated cirrhosis; HCC, hepatocellular carcinoma; LT, liver transplantation; HR, hazard ratio; CI, confidence interval.

© 2025 Shi Y et al. *JAMA Network Open*.

eTable 7. Association Between Sex and Adverse Liver Events in Subgroup by Presence of Hepatic Decompensation

	Compensated cirrhosis				Decompensated cirrhosis			
	**No. of patients**	**No. of events**	**HR (95% CI)**	***P* value**	**No. of patients**	**No. of events**	**HR (95% CI)**	***P* value**
**HCC**								
Male	73485	359	1.84 (1.56, 2.17)	<0.001	48376	2046	2.03 (1.88, 2.19)	<0.001
Fema	79060	225	Ref		45973	1041	Ref	
**LT**								
Male	73395	265	1.65 (1.37, 2.00)	<0.001	47536	3196	1.53 (1.45, 1.62)	<0.001
Fema	78992	183	Ref		45417	2156	Ref	

Statistical analysis was performed by a Cox-proportional hazard model.
Abbreviation: HCC, hepatocellular carcinoma; LT, liver transplantation; HR, hazard ratio; CI, confidence interval.

© 2025 Shi Y et al. *JAMA Network Open*.

eTable 8. Association Between Sex and Adverse Liver Events in Subgroup by the Time Periods of Cirrhosis Diagnosis

	Before or in 2014				After 2014			
	**No. of patients**	**No. of events**	**HR (95% CI)**	***P* value**	**No. of patients**	**No. of events**	**HR (95% CI)**	***P* value**
**DC**								
Male	66794	11249	1.19 (1.16, 1.22)	<0.001	41996	4296	1.11 (1.06, 1.15)	<0.001
Female	70827	10673	Ref		42507	4037	Ref	
**HCC**								
Male	76204	1926	2.16 (2.00, 2.33)	<0.001	45657	479	1.93 (1.66, 2.24)	<0.001
Female	79240	1006	Ref		45793	260	Ref	
**LT**								
Male	75579	2556	1.68 (1.58, 1.80)	<0.001	45352	905	1.49 (1.35, 1.65)	<0.001
Female	78828	1706	Ref		45581	633	Ref	

Statistical analysis was performed by a Cox-proportional hazard model.
Abbreviation: DC, decompensated cirrhosis; HCC, hepatocellular carcinoma; LT, liver transplantation; HR, hazard ratio; CI, confidence interval.

© 2025 Shi Y et al. *JAMA Network Open*.

eTable 9. Association Between Sex and Liver Transplantation in Subgroup by HCC and Non-HCC

	No. of patients	No. of events	HR (95% CI)	*P* value
**LT for HCC**				
Male	120931	766	2.42 (2.14, 2.75)	<0.001
Female	124409	356	Ref	
**LT for non-HCC**				
Male	120931	2695	1.48 (1.40, 1.57)	<0.001
Female	124409	1983	Ref	

Statistical analysis was performed by a Cox-proportional hazard model.

Abbreviation: HCC, hepatocellular carcinoma; LT, liver transplantation; HR, hazard ratio; CI, confidence interval.

© 2025 Shi Y et al. *JAMA Network Open*.

eFigure 1. Cumulative Incidence of DC (A), HCC (B), and LT (C) in Patients With Cirrhosis, by Sex and Age

Survival probabilities were estimated by Kaplan–Meier approach and compared by log-rank test.

DC, decompensated cirrhosis; HCC, hepatocellular carcinoma; LT, liver transplantation.

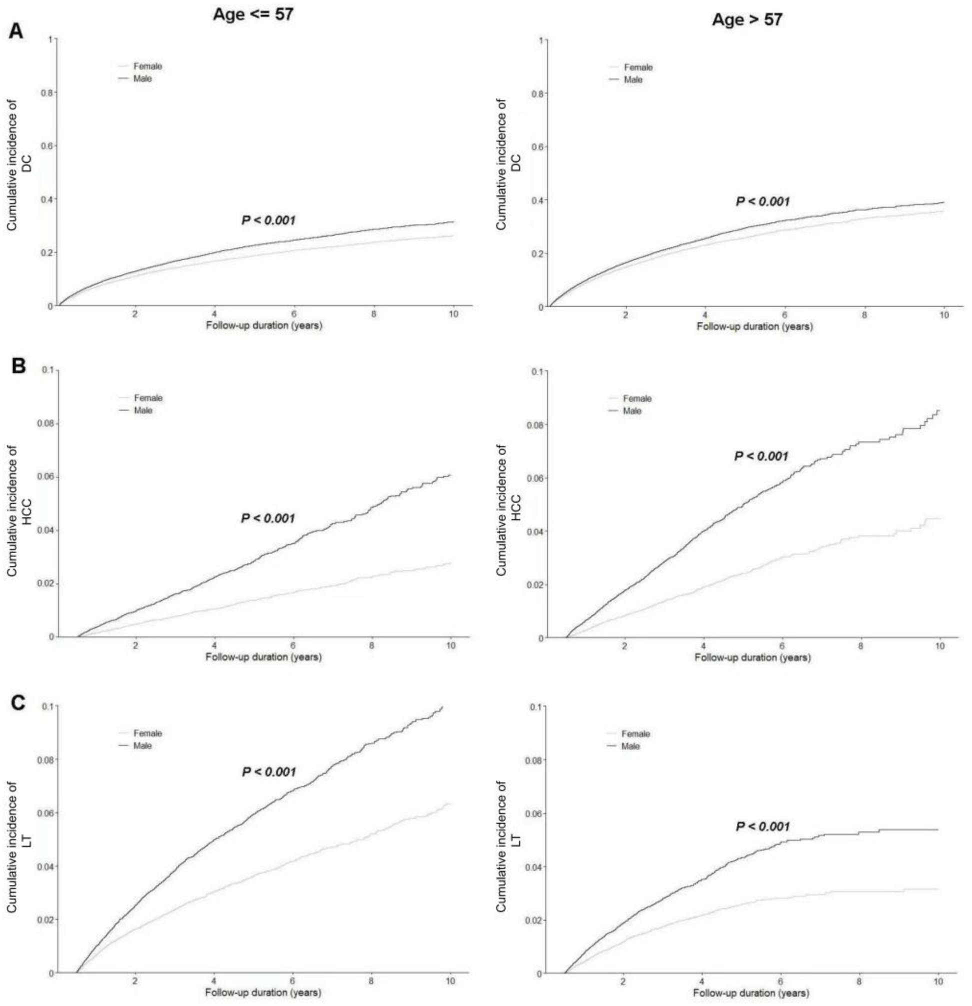

© 2025 Shi Y et al. *JAMA Network Open*.

eFigure 2. Cumulative Incidence of HCC (A) and LT (B) in Patients With Cirrhosis, by Sex and Baseline Decompensation

Survival probabilities were estimated by Kaplan–Meier approach and compared by log-rank test. HCC, hepatocellular carcinoma; LT, liver transplantation.

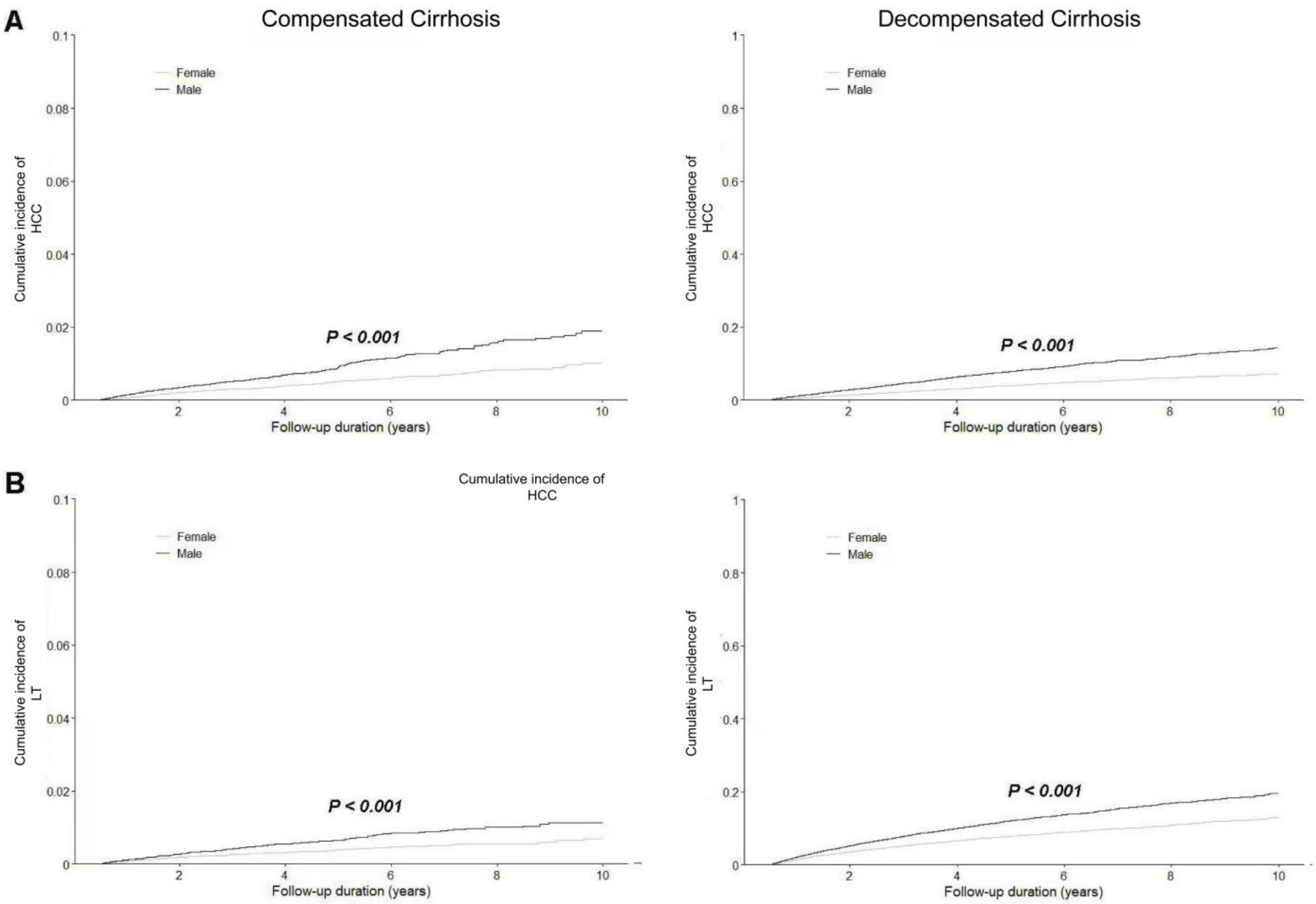

© 2025 Shi Y et al. *JAMA Network Open*.
