# Supplementary material for: Sex Differences in Risk of Adverse Liver Events in Patients With Cirrhosis
Source: JAMA Netw Open. 2025 Jul 28;8(7):e2523674. doi: 10.1001/jamanetworkopen.2025.23674 (PMC12305389; doi:10.1001/jamanetworkopen.2025.23674)
Supplement: Supplement 2. — Data Sharing Statement [file jamanetwopen-e2523674-s002.pdf]

Data Sharing Statement

Shi. Sex Differences in Risk of Adverse Liver Events in Patients With Cirrhosis. *JAMA Netw Open*. Published July 28, 2025. doi:10.1001/jamanetworkopen.2025.23674

Data
Data available: No
